# Supplementary material for: Successional Shifts in Soil C:N:P stoichiometry and its environmental controls during forest restoration in Huize County, Qujing, Yunnan Province
Source: PLoS One. 2026 Aug 21;21(8):e0356261. doi: 10.1371/journal.pone.0356261 (PMC13496410; doi:10.1371/journal.pone.0356261)
Supplement: Supplementary file 2 [file pone.0356261.s004.zip › Data and Code/RF/mixed conifer–broadleaf forest/figures/PDP_N.P_S.NO.N.pdf]

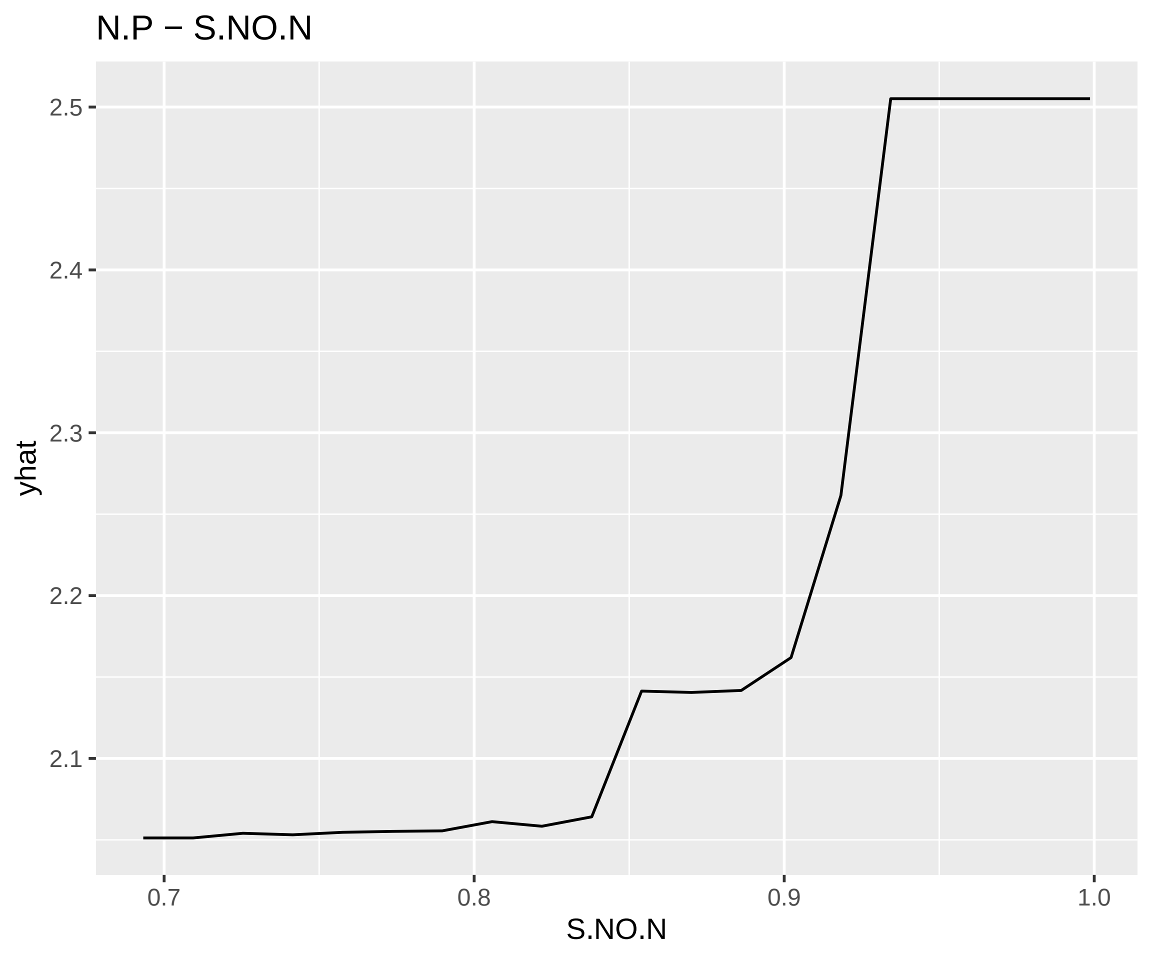
N.P − S.NO.N
yhat
2.5
2.4
2.3
2.2
2.1
0.7
0.8
0.9
1.0
S.NO.N
